# Methyl 2-[2-((*Z*)-{1-*trans*-[2-(4-fluoro-3-methyl­phen­yl)-2-methyl­cyclo­prop­yl]ethyl­idene}amino­oxymeth­yl)phen­yl]-2-[(*E*)-methoxy­imino]­acetate

**DOI:** 10.1107/S1600536809009064

**Published:** 2009-03-19

**Authors:** Chunfeng Song, Ronald Ross, Steven Shaber, Bin Li

**Affiliations:** a Medical College of Chifeng University, Chifeng 024000, People’s Republic of China; b Dow AgroSciences, 9330 Zionsville Road, Indianapolis, Indiana 46268, USA; c Agrochemicals Division, Shenyang Research Institute of Chemical Industry, Shenyang 110021, People’s Republic of China

## Abstract

The title compound, C_24_H_27_FN_2_O_4_, is an important inter­mediate in the synthesis of fungicidal strobilurin-type compounds. In the crystal structure, the oxime bond attached to the cyclo­propane ring adopts a *Z* configuration, while the oxime bond attached to the benzene ring adopts an *E* configuration. The fluoro­methyl­phenyl group adopts a *trans* configuration with respect to the remainder of the mol­ecule, and its mean plane forms a dihedral angle of 56.1 (1)° with the plane of the cyclo­propane ring.

## Related literature

For synthesis details and related literature, see: Li *et al.* (2008 ▶); Ross *et al.* (2001 ▶).
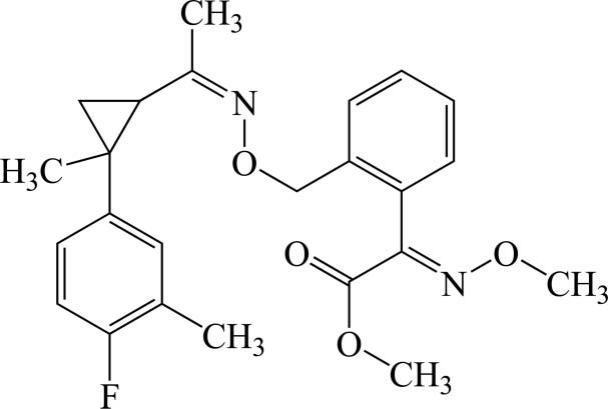


## Experimental

### 

#### Crystal data

C_24_H_27_FN_2_O_4_
*M* *_r_* = 426.48
Triclinic, 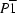

*a* = 7.819 (3) Å
*b* = 10.901 (4) Å
*c* = 14.565 (5) Å
α = 105.134 (4)°
β = 92.014 (4)°
γ = 104.718 (4)°
*V* = 1152.3 (7) Å^3^
*Z* = 2
Mo *K*α radiation
μ = 0.09 mm^−1^
*T* = 293 K
0.34 × 0.30 × 0.24 mm

#### Data collection

Bruker APEXII CCD diffractometer
Absorption correction: multi-scan (*SADABS*; Sheldrick, 2003 ▶) *T*_min_ = 0.754, *T*_max_ = 0.979
6297 measured reflections
4026 independent reflections
2977 reflections with *I* > 2σ(*I*)
*R*_int_ = 0.015

#### Refinement

*R*[*F* ^2^ > 2σ(*F* ^2^)] = 0.046
*wR*(*F* ^2^) = 0.140
*S* = 1.05
4026 reflections
285 parameters
H-atom parameters constrained
Δρ_max_ = 0.26 e Å^−3^
Δρ_min_ = −0.15 e Å^−3^

### 

Data collection: *APEX2* (Bruker, 2004 ▶); cell refinement: *SAINT* (Bruker, 2003 ▶); data reduction: *SAINT*; program(s) used to solve structure: *SHELXS97* (Sheldrick, 2008 ▶); program(s) used to refine structure: *SHELXL97* (Sheldrick, 2008 ▶); molecular graphics: *SHELXTL* (Sheldrick, 2008 ▶); software used to prepare material for publication: *SHELXTL*.

## Supplementary Material

Crystal structure: contains datablocks I, global. DOI: 10.1107/S1600536809009064/bi2355sup1.cif

Structure factors: contains datablocks I. DOI: 10.1107/S1600536809009064/bi2355Isup2.hkl

Additional supplementary materials:  crystallographic information; 3D view; checkCIF report

## Figures and Tables

**Fig. 1.**
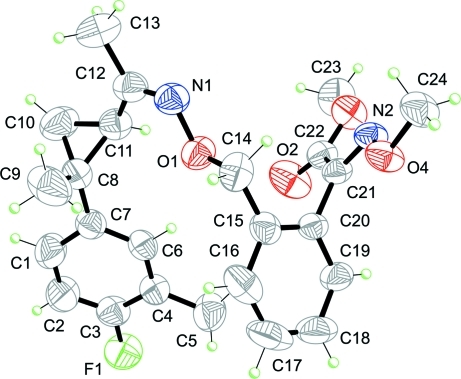
Molecular structure of the title compound with 30% probability displacement ellipsoids for non-H atoms.

## supplementary crystallographic information

### Comment

Some cyclopropyl-containing Strobilurin-type compounds have been reported as a new class of fungicides characterized by their broad spectrum and high levels of fungicidal activity (Ross *et al.*, 2001; Li *et al.*, 2008). The title compound is an intermediate for preparing such compounds and the crystal structure is important to identify the configuration of possible products.

### Experimental

The title compound was prepared according to a published procedure (Li *et al.*, 2008) as follows: trans 2-(4-fluoro-3-methylphenyl)-2-methyl-1-acetylcyclopropane (26.8 g, 13 mmol) and methyl *E*-2-(aminooxymethyl)phenyl glyoxylate *O*-methyloxime (34.0 g, 14.3 mmol) were added into methanol (500 ml) and acetic acid (0.7 ml). The mixture was stirred for 14 h and washed with water and brine, dried by anhydrous magnesium sulfate, then evaporated to give an oily product. This product was seperated with silica gel chromatography to give major product A (90%) and minor product B (10%). The product B was dissolved in acetone and left to stand at room temperature. Block crystals suitable for X-ray analyses were obtained.

^1^H NMR(CDCl_3_): δ 7.78–7.81 (m, 1H), 7.523–7.492 (m, 1H), 7.412–7.382 (m, 2H), 7.212–7.195 (m, 1H), 7.177–7.035 (m, 2H), 6.785–6.753 (t, 1H), 5.044–5.038 (d, 2H), 3.948 (s, 3H), 3.705 (s, 3H), 2.055–1.986 (m, 4H), 1.868 (s, 3H), 1.205–1.189 (m, 4H).

### Refinement

Although all H atoms were visible in difference Fourier maps, they were finally placed in geometrically calculated positions, with C—H distances in the range 0.93–0.98 Å, and included in the final refinement in the riding model approximation, with *U*_iso_(H) = 1.2 or 1.5*U*_eq_(C).

### Crystal data

**Table:**

C_24_H_27_FN_2_O_4_	*Z* = 2
*M**_r_* = 426.48	*F*(000) = 452
Triclinic, *P*1	*D*_x_ = 1.229 Mg m^−^^3^
Hall symbol: -P 1	Mo *K*α radiation, λ = 0.71073 Å
*a* = 7.819 (3) Å	Cell parameters from 2258 reflections
*b* = 10.901 (4) Å	θ = 2.7–25.2°
*c* = 14.565 (5) Å	µ = 0.09 mm^−^^1^
α = 105.134 (4)°	*T* = 293 K
β = 92.014 (4)°	Block, colorless
γ = 104.718 (4)°	0.34 × 0.30 × 0.24 mm
*V* = 1152.3 (7) Å^3^	

### Data collection

**Table:**

Bruker APEXII CCD diffractometer	4026 independent reflections
Radiation source: fine-focus sealed tube	2977 reflections with *I* > 2σ(*I*)
graphite	*R*_int_ = 0.015
φ and ω scans	θ_max_ = 25.0°, θ_min_ = 2.0°
Absorption correction: multi-scan (*SADABS*; Sheldrick, 2003)	*h* = −9→9
*T*_min_ = 0.754, *T*_max_ = 0.979	*k* = −9→12
6297 measured reflections	*l* = −17→17

### Refinement

**Table:**

Refinement on *F*^2^	Primary atom site location: structure-invariant direct methods
Least-squares matrix: full	Secondary atom site location: difference Fourier map
*R*[*F*^2^ > 2σ(*F*^2^)] = 0.046	Hydrogen site location: inferred from neighbouring sites
*wR*(*F*^2^) = 0.140	H-atom parameters constrained
*S* = 1.05	*w* = 1/[σ^2^(*F*_o_^2^) + (0.0682*P*)^2^ + 0.2537*P*] where *P* = (*F*_o_^2^ + 2*F*_c_^2^)/3
4026 reflections	(Δ/σ)_max_ < 0.001
285 parameters	Δρ_max_ = 0.26 e Å^−^^3^
0 restraints	Δρ_min_ = −0.15 e Å^−^^3^

### Special details

**Table:**

Geometry. All e.s.d.'s (except the e.s.d. in the dihedral angle between two l.s. planes) are estimated using the full covariance matrix. The cell e.s.d.'s are taken into account individually in the estimation of e.s.d.'s in distances, angles and torsion angles; correlations between e.s.d.'s in cell parameters are only used when they are defined by crystal symmetry. An approximate (isotropic) treatment of cell e.s.d.'s is used for estimating e.s.d.'s involving l.s. planes.
Refinement. Refinement of *F*^2^ against ALL reflections. The weighted *R*-factor *wR* and goodness of fit *S* are based on *F*^2^, conventional *R*-factors *R* are based on *F*, with *F* set to zero for negative *F*^2^. The threshold expression of *F*^2^ > σ(*F*^2^) is used only for calculating *R*-factors(gt) *etc*. and is not relevant to the choice of reflections for refinement. *R*-factors based on *F*^2^ are statistically about twice as large as those based on *F*, and *R*- factors based on ALL data will be even larger.

### Fractional atomic coordinates and isotropic or equivalent isotropic displacement parameters (Å^2^)

**Table:**

	*x*	*y*	*z*	*U*_iso_*/*U*_eq_	
F1	1.35756 (18)	0.82907 (15)	0.97952 (11)	0.0942 (5)	
O1	0.56929 (18)	0.50914 (14)	0.67273 (10)	0.0625 (4)	
O2	0.6063 (2)	0.39143 (18)	0.88366 (11)	0.0903 (6)	
O3	0.33762 (19)	0.25037 (15)	0.86384 (10)	0.0680 (4)	
O4	0.41061 (16)	0.08197 (13)	0.59611 (9)	0.0600 (4)	
N1	0.4299 (2)	0.55373 (18)	0.63920 (12)	0.0604 (4)	
N2	0.37516 (19)	0.14127 (15)	0.68779 (10)	0.0509 (4)	
C1	1.0499 (3)	0.9394 (2)	0.8529 (2)	0.0836 (7)	
H1	1.0399	1.0172	0.8411	0.100*	
C2	1.1977 (3)	0.9404 (2)	0.9074 (2)	0.0879 (8)	
H2	1.2875	1.0183	0.9325	0.105*	
C3	1.2108 (3)	0.8262 (2)	0.92420 (16)	0.0680 (6)	
C4	1.0856 (3)	0.7074 (2)	0.88688 (14)	0.0588 (5)	
C5	1.1107 (4)	0.5818 (3)	0.9027 (2)	0.0895 (8)	
H5A	1.2018	0.5563	0.8658	0.134*	
H5B	1.0012	0.5131	0.8829	0.134*	
H5C	1.1452	0.5958	0.9693	0.134*	
C6	0.9380 (3)	0.7098 (2)	0.83200 (14)	0.0558 (5)	
H6	0.8506	0.6309	0.8053	0.067*	
C7	0.9156 (3)	0.8237 (2)	0.81544 (15)	0.0590 (5)	
C8	0.7563 (3)	0.8232 (2)	0.75450 (16)	0.0637 (5)	
C9	0.7882 (4)	0.8208 (4)	0.6527 (2)	0.1044 (10)	
H9A	0.8837	0.8960	0.6523	0.157*	
H9B	0.6821	0.8235	0.6189	0.157*	
H9C	0.8192	0.7413	0.6219	0.157*	
C10	0.6289 (3)	0.8960 (3)	0.8011 (2)	0.0918 (8)	
H10A	0.6542	0.9400	0.8691	0.110*	
H10B	0.5744	0.9411	0.7642	0.110*	
C11	0.5746 (3)	0.7483 (2)	0.77037 (15)	0.0624 (5)	
H11	0.5744	0.7077	0.8230	0.075*	
C12	0.4369 (3)	0.6710 (2)	0.68858 (15)	0.0609 (5)	
C13	0.2966 (3)	0.7290 (3)	0.65974 (19)	0.0849 (7)	
H13A	0.3490	0.7967	0.6302	0.127*	
H13B	0.2447	0.7664	0.7153	0.127*	
H13C	0.2061	0.6610	0.6152	0.127*	
C14	0.5777 (3)	0.3937 (2)	0.60237 (15)	0.0659 (6)	
H14A	0.6049	0.4141	0.5427	0.079*	
H14B	0.4643	0.3271	0.5910	0.079*	
C15	0.7210 (3)	0.34381 (19)	0.63878 (14)	0.0552 (5)	
C16	0.8912 (3)	0.3751 (2)	0.61019 (18)	0.0790 (7)	
H16	0.9156	0.4278	0.5688	0.095*	
C17	1.0237 (3)	0.3291 (3)	0.6423 (2)	0.0924 (9)	
H17	1.1361	0.3505	0.6219	0.111*	
C18	0.9913 (3)	0.2521 (3)	0.7040 (2)	0.0822 (8)	
H18	1.0807	0.2205	0.7251	0.099*	
C19	0.8259 (3)	0.2222 (2)	0.73409 (16)	0.0629 (5)	
H19	0.8041	0.1715	0.7770	0.075*	
C20	0.6902 (2)	0.26630 (17)	0.70163 (13)	0.0482 (4)	
C21	0.5128 (2)	0.22899 (18)	0.73554 (12)	0.0460 (4)	
C22	0.4921 (3)	0.3000 (2)	0.83565 (13)	0.0536 (5)	
C23	0.3080 (4)	0.3151 (3)	0.95956 (16)	0.0839 (7)	
H23A	0.3990	0.3128	1.0046	0.126*	
H23B	0.1937	0.2702	0.9735	0.126*	
H23C	0.3116	0.4052	0.9642	0.126*	
C24	0.2488 (3)	−0.0023 (2)	0.54163 (15)	0.0660 (6)	
H24A	0.1929	−0.0644	0.5749	0.099*	
H24B	0.2740	−0.0491	0.4803	0.099*	
H24C	0.1707	0.0499	0.5329	0.099*	

### Atomic displacement parameters (Å^2^)

**Table:**

	*U*^11^	*U*^22^	*U*^33^	*U*^12^	*U*^13^	*U*^23^
F1	0.0727 (9)	0.0917 (10)	0.1010 (10)	0.0112 (7)	−0.0317 (8)	0.0138 (8)
O1	0.0619 (8)	0.0581 (8)	0.0631 (8)	0.0179 (7)	−0.0104 (7)	0.0099 (7)
O2	0.0780 (11)	0.0935 (12)	0.0624 (9)	−0.0017 (10)	0.0000 (8)	−0.0159 (9)
O3	0.0682 (9)	0.0725 (10)	0.0519 (8)	0.0143 (8)	0.0142 (7)	0.0015 (7)
O4	0.0439 (7)	0.0660 (9)	0.0507 (7)	0.0069 (6)	0.0036 (6)	−0.0089 (6)
N1	0.0528 (10)	0.0679 (12)	0.0589 (10)	0.0149 (8)	−0.0040 (8)	0.0179 (9)
N2	0.0423 (8)	0.0574 (9)	0.0468 (8)	0.0151 (7)	0.0040 (7)	0.0025 (7)
C1	0.0752 (16)	0.0552 (13)	0.111 (2)	0.0105 (12)	−0.0123 (14)	0.0179 (13)
C2	0.0710 (16)	0.0578 (15)	0.113 (2)	0.0013 (12)	−0.0212 (14)	0.0066 (14)
C3	0.0567 (12)	0.0704 (15)	0.0662 (13)	0.0114 (11)	−0.0096 (10)	0.0086 (11)
C4	0.0593 (12)	0.0604 (12)	0.0536 (11)	0.0124 (10)	0.0020 (9)	0.0150 (9)
C5	0.0882 (18)	0.0775 (17)	0.1009 (19)	0.0139 (14)	−0.0201 (15)	0.0343 (15)
C6	0.0532 (11)	0.0544 (12)	0.0532 (11)	0.0056 (9)	0.0018 (9)	0.0133 (9)
C7	0.0538 (11)	0.0591 (12)	0.0617 (12)	0.0129 (9)	0.0047 (9)	0.0152 (10)
C8	0.0556 (12)	0.0641 (13)	0.0752 (14)	0.0126 (10)	0.0068 (10)	0.0293 (11)
C9	0.0740 (17)	0.153 (3)	0.0969 (19)	0.0095 (17)	0.0048 (14)	0.074 (2)
C10	0.0781 (16)	0.0698 (16)	0.118 (2)	0.0295 (13)	−0.0085 (15)	0.0025 (15)
C11	0.0602 (12)	0.0647 (13)	0.0608 (12)	0.0187 (10)	0.0047 (10)	0.0138 (10)
C12	0.0527 (11)	0.0747 (15)	0.0586 (12)	0.0183 (10)	0.0055 (9)	0.0231 (11)
C13	0.0769 (16)	0.107 (2)	0.0785 (16)	0.0456 (15)	0.0001 (13)	0.0204 (14)
C14	0.0794 (15)	0.0581 (13)	0.0561 (12)	0.0167 (11)	−0.0040 (10)	0.0123 (10)
C15	0.0523 (11)	0.0469 (11)	0.0556 (11)	0.0063 (8)	0.0017 (9)	0.0035 (9)
C16	0.0671 (15)	0.0739 (15)	0.0773 (15)	−0.0045 (12)	0.0139 (12)	0.0112 (12)
C17	0.0399 (12)	0.096 (2)	0.111 (2)	0.0015 (12)	0.0135 (13)	−0.0070 (17)
C18	0.0427 (12)	0.0842 (17)	0.105 (2)	0.0210 (12)	−0.0052 (12)	−0.0011 (15)
C19	0.0465 (11)	0.0586 (12)	0.0778 (14)	0.0172 (9)	−0.0082 (10)	0.0082 (10)
C20	0.0395 (9)	0.0443 (10)	0.0517 (10)	0.0107 (8)	−0.0021 (8)	−0.0005 (8)
C21	0.0400 (9)	0.0493 (10)	0.0470 (10)	0.0164 (8)	−0.0018 (8)	0.0071 (8)
C22	0.0516 (11)	0.0551 (11)	0.0494 (10)	0.0159 (9)	−0.0017 (9)	0.0061 (9)
C23	0.1056 (19)	0.0876 (18)	0.0553 (13)	0.0333 (15)	0.0254 (13)	0.0059 (12)
C24	0.0537 (12)	0.0676 (13)	0.0585 (12)	0.0052 (10)	−0.0089 (9)	−0.0006 (10)

### Geometric parameters (Å, °)

**Table:**

F1—C3	1.368 (2)	C10—C11	1.496 (3)
O1—C14	1.418 (2)	C10—H10A	0.970
O1—N1	1.421 (2)	C10—H10B	0.970
O2—C22	1.192 (2)	C11—C12	1.487 (3)
O3—C22	1.319 (2)	C11—H11	0.980
O3—C23	1.445 (3)	C12—C13	1.496 (3)
O4—N2	1.3944 (19)	C13—H13A	0.960
O4—C24	1.425 (2)	C13—H13B	0.960
N1—C12	1.279 (3)	C13—H13C	0.960
N2—C21	1.278 (2)	C14—C15	1.501 (3)
C1—C2	1.375 (3)	C14—H14A	0.970
C1—C7	1.385 (3)	C14—H14B	0.970
C1—H1	0.930	C15—C20	1.388 (3)
C2—C3	1.358 (3)	C15—C16	1.394 (3)
C2—H2	0.930	C16—C17	1.376 (4)
C3—C4	1.371 (3)	C16—H16	0.930
C4—C6	1.390 (3)	C17—C18	1.371 (4)
C4—C5	1.505 (3)	C17—H17	0.930
C5—H5A	0.960	C18—C19	1.368 (3)
C5—H5B	0.960	C18—H18	0.930
C5—H5C	0.960	C19—C20	1.387 (3)
C6—C7	1.377 (3)	C19—H19	0.930
C6—H6	0.930	C20—C21	1.483 (3)
C7—C8	1.502 (3)	C21—C22	1.497 (3)
C8—C10	1.500 (3)	C23—H23A	0.960
C8—C9	1.507 (3)	C23—H23B	0.960
C8—C11	1.508 (3)	C23—H23C	0.960
C9—H9A	0.960	C24—H24A	0.960
C9—H9B	0.960	C24—H24B	0.960
C9—H9C	0.960	C24—H24C	0.960
			
C14—O1—N1	107.55 (14)	C8—C11—H11	114.3
C22—O3—C23	116.14 (17)	N1—C12—C11	123.38 (19)
N2—O4—C24	109.21 (14)	N1—C12—C13	116.2 (2)
C12—N1—O1	111.23 (16)	C11—C12—C13	120.5 (2)
C21—N2—O4	111.21 (14)	C12—C13—H13A	109.5
C2—C1—C7	120.8 (2)	C12—C13—H13B	109.5
C2—C1—H1	119.6	H13A—C13—H13B	109.5
C7—C1—H1	119.6	C12—C13—H13C	109.5
C3—C2—C1	119.1 (2)	H13A—C13—H13C	109.5
C3—C2—H2	120.4	H13B—C13—H13C	109.5
C1—C2—H2	120.4	O1—C14—C15	107.78 (16)
C2—C3—F1	118.4 (2)	O1—C14—H14A	110.2
C2—C3—C4	123.2 (2)	C15—C14—H14A	110.2
F1—C3—C4	118.4 (2)	O1—C14—H14B	110.2
C3—C4—C6	116.2 (2)	C15—C14—H14B	110.2
C3—C4—C5	121.4 (2)	H14A—C14—H14B	108.5
C6—C4—C5	122.4 (2)	C20—C15—C16	117.8 (2)
C4—C5—H5A	109.5	C20—C15—C14	121.82 (18)
C4—C5—H5B	109.5	C16—C15—C14	120.4 (2)
H5A—C5—H5B	109.5	C17—C16—C15	121.1 (3)
C4—C5—H5C	109.5	C17—C16—H16	119.5
H5A—C5—H5C	109.5	C15—C16—H16	119.5
H5B—C5—H5C	109.5	C18—C17—C16	120.5 (2)
C7—C6—C4	122.83 (19)	C18—C17—H17	119.7
C7—C6—H6	118.6	C16—C17—H17	119.7
C4—C6—H6	118.6	C19—C18—C17	119.2 (2)
C6—C7—C1	117.8 (2)	C19—C18—H18	120.4
C6—C7—C8	121.80 (19)	C17—C18—H18	120.4
C1—C7—C8	120.3 (2)	C18—C19—C20	121.0 (2)
C10—C8—C7	118.3 (2)	C18—C19—H19	119.5
C10—C8—C9	118.5 (2)	C20—C19—H19	119.5
C7—C8—C9	114.35 (19)	C19—C20—C15	120.29 (18)
C10—C8—C11	59.65 (15)	C19—C20—C21	118.64 (18)
C7—C8—C11	118.65 (18)	C15—C20—C21	121.07 (16)
C9—C8—C11	116.8 (2)	N2—C21—C20	126.38 (16)
C8—C9—H9A	109.5	N2—C21—C22	116.44 (16)
C8—C9—H9B	109.5	C20—C21—C22	117.18 (15)
H9A—C9—H9B	109.5	O2—C22—O3	124.65 (18)
C8—C9—H9C	109.5	O2—C22—C21	122.16 (19)
H9A—C9—H9C	109.5	O3—C22—C21	113.20 (16)
H9B—C9—H9C	109.5	O3—C23—H23A	109.5
C11—C10—C8	60.42 (15)	O3—C23—H23B	109.5
C11—C10—H10A	117.7	H23A—C23—H23B	109.5
C8—C10—H10A	117.7	O3—C23—H23C	109.5
C11—C10—H10B	117.7	H23A—C23—H23C	109.5
C8—C10—H10B	117.7	H23B—C23—H23C	109.5
H10A—C10—H10B	114.9	O4—C24—H24A	109.5
C12—C11—C10	122.1 (2)	O4—C24—H24B	109.5
C12—C11—C8	121.18 (19)	H24A—C24—H24B	109.5
C10—C11—C8	59.93 (16)	O4—C24—H24C	109.5
C12—C11—H11	114.3	H24A—C24—H24C	109.5
C10—C11—H11	114.3	H24B—C24—H24C	109.5
			
C14—O1—N1—C12	−166.73 (18)	C10—C11—C12—N1	154.4 (2)
C24—O4—N2—C21	−172.57 (17)	C8—C11—C12—N1	82.6 (3)
C7—C1—C2—C3	0.0 (4)	C10—C11—C12—C13	−25.6 (3)
C1—C2—C3—F1	−179.2 (2)	C8—C11—C12—C13	−97.4 (3)
C1—C2—C3—C4	2.0 (4)	N1—O1—C14—C15	−178.28 (16)
C2—C3—C4—C6	−1.9 (4)	O1—C14—C15—C20	82.5 (2)
F1—C3—C4—C6	179.34 (18)	O1—C14—C15—C16	−97.2 (2)
C2—C3—C4—C5	176.4 (3)	C20—C15—C16—C17	0.8 (3)
F1—C3—C4—C5	−2.4 (3)	C14—C15—C16—C17	−179.4 (2)
C3—C4—C6—C7	−0.3 (3)	C15—C16—C17—C18	−0.5 (4)
C5—C4—C6—C7	−178.6 (2)	C16—C17—C18—C19	−0.6 (4)
C4—C6—C7—C1	2.2 (3)	C17—C18—C19—C20	1.3 (3)
C4—C6—C7—C8	178.95 (19)	C18—C19—C20—C15	−1.0 (3)
C2—C1—C7—C6	−2.1 (4)	C18—C19—C20—C21	179.03 (19)
C2—C1—C7—C8	−178.9 (2)	C16—C15—C20—C19	−0.1 (3)
C6—C7—C8—C10	115.1 (2)	C14—C15—C20—C19	−179.79 (17)
C1—C7—C8—C10	−68.2 (3)	C16—C15—C20—C21	179.88 (17)
C6—C7—C8—C9	−98.0 (3)	C14—C15—C20—C21	0.1 (3)
C1—C7—C8—C9	78.7 (3)	O4—N2—C21—C20	−0.3 (3)
C6—C7—C8—C11	46.2 (3)	O4—N2—C21—C22	−179.87 (15)
C1—C7—C8—C11	−137.1 (2)	C19—C20—C21—N2	−104.2 (2)
C7—C8—C10—C11	−108.4 (2)	C15—C20—C21—N2	75.9 (3)
C9—C8—C10—C11	106.1 (2)	C19—C20—C21—C22	75.4 (2)
C8—C10—C11—C12	−110.1 (2)	C15—C20—C21—C22	−104.5 (2)
C10—C8—C11—C12	111.6 (3)	C23—O3—C22—O2	1.0 (3)
C7—C8—C11—C12	−140.6 (2)	C23—O3—C22—C21	−179.41 (17)
C9—C8—C11—C12	2.7 (3)	N2—C21—C22—O2	−173.2 (2)
C7—C8—C11—C10	107.8 (2)	C20—C21—C22—O2	7.2 (3)
C9—C8—C11—C10	−108.8 (3)	N2—C21—C22—O3	7.2 (2)
O1—N1—C12—C11	−0.1 (3)	C20—C21—C22—O3	−172.41 (16)
O1—N1—C12—C13	179.85 (18)		
